# Evolution of a novel subfamily of nuclear receptors with members that each contain two DNA binding domains

**DOI:** 10.1186/1471-2148-7-27

**Published:** 2007-02-23

**Authors:** Wenjie Wu, Edward G Niles, Hirohisa Hirai, Philip T LoVerde

**Affiliations:** 1 Department of Microbiology and Immunology, School of Medicine and Biomedical Science, State University of New York, Buffalo, NY 14214, USA; 2 Primate Research Institute, Kyoto University, Inuyama, Japan; 3 Southwest Foundation for Biomedical Research, PO Box 760549, San Antonio, Texas, 78245-0594, USA

## Abstract

**Background:**

Nuclear receptors (NRs) are important transcriptional modulators in metazoans which regulate transcription through binding to the promoter region of their target gene by the DNA binding domain (DBD) and activation or repression of mRNA synthesis through co-regulators bound to the ligand binding domain (LBD). NRs typically have a single DBD with a LBD.

**Results:**

Three nuclear receptors named 2DBD-NRs, were identified from the flatworm *Schistosoma mansoni *that each possess a novel set of two DBDs in tandem with a LBD. They represent a novel NR modular structure: A/B-DBD-DBD-hinge-LBD. The 2DBD-NRs form a new subfamily of NRs, VII. By database mining, 2DBD-NR genes from other flatworm species (*Schmidtea mediterranea *and *Dugesia japonica*), from Mollusks (*Lottia gigantean*) and from arthropods (*Daphnia pulex*) were also identified. All 2DBD-NRs possess a P-box sequence of CEACKK in the first DBD, which is unique to 2DBD-NRs, and a P-box sequence of CEGCKG in the second DBD. Phylogenetic analyses of both DBD and ligand binding domain sequences showed that 2DBD-NR genes originate from a common two DBD-containing ancestor gene. A single 2DBD-NR orthologue was found in Arthropoda, Platyhelminths and Mollusca. Subsequent 2DBD-NR gene evolution in Mollusks and Platyhelminths involved gene duplication. Chromosome localization of *S. mansoni *2DBD-NR genes by Fluorescent *in situ *hybridization (FISH) suggests that 2DBD-NR genes duplicated on different chromosomes in the Platyhelminths. Dimerization of Sm2DBDα indicates that 2DBD-NRs may act as homodimers, suggesting either that two repeats of a half-site are necessary for each DBD of 2DBD-NRs to bind to its target gene, or that each 2DBD-NR can recognize multiple sites.

**Conclusion:**

*2DBD-NRs *share a common ancestor gene which possessed an extra DBD that likely resulted from a recombination event. After the split of the Arthropods, Mollusks and Platyhelminths, 2*DBD-NR *underwent a recent duplication in a common ancestor of Mollusks, while two rounds of duplication occurred in a common ancestor of the Platyhelminths. This demonstrates that certain NR gene underwent recent duplication in Prostostome lineages after the split of the Prostostomia and Deuterostomia.

## Background

Nuclear receptors (NR) regulate homeostasis, differentiation, metamorphosis and reproduction in metazoans. Members of the nuclear receptor superfamily are characterized by a modular structure. Typical NRs contain an N-terminal A/B domain, a C domain (DNA binding domain, DBD), a D domain (hinge) and an E domain (ligand binding domain, LBD). The most conserved region in NRs is the DBD, which contains two zinc finger motifs (CI and CII). There is a conserved sequence element in the DBD, called the P-box, which confers target DNA binding specificity. Another moderately conserved region is the LBD [1-3]. Two highly conserved regions are present within the LBD. The first region is called the signature sequence of LBD (Tau, Tτ), from the C-terminus of helix 3 to the middle of helix 4 [1,4]. The second conserved region is helix 12 which contains the activation function core motif (AF2-AD) that is required for transcriptional activation and co-regulator recruitment. NRs regulate transcription through binding to the promoter region of their target gene by the DBD and activation or repression of mRNA synthesis through co-regulators bound to the LBD [5].

Recently, we isolated three partial cDNAs of nuclear receptors which contain two DBDs in the flatworm *Schistosoma mansoni *[6]. Typical NRs only have a single DBD with a LBD, unusual nuclear receptors are known only to have one DBD without a LBD [7-10] or to posses a LBD without a DBD [11,12]. To determine the modular structure of these novel nuclear receptors (that is, whether they contain a LBD), cDNAs encoding the entire open reading frame (ORF) of Sm2DBD-NRs were isolated. By data mining, additional *2DBD-NRs *were identified in species of Mollusca, Arthropoda and other species of Platyhelminths. The phylogenetic relationship of *2DBD-NRs *was constructed, the origin of *2DBD-NRs *and their role in understanding metazoan phylogeny is discussed.

## Results and Discussion

### A novel NR modular structure: A/B-DBD-DBD-hinge-LBD

**Figure 1:**
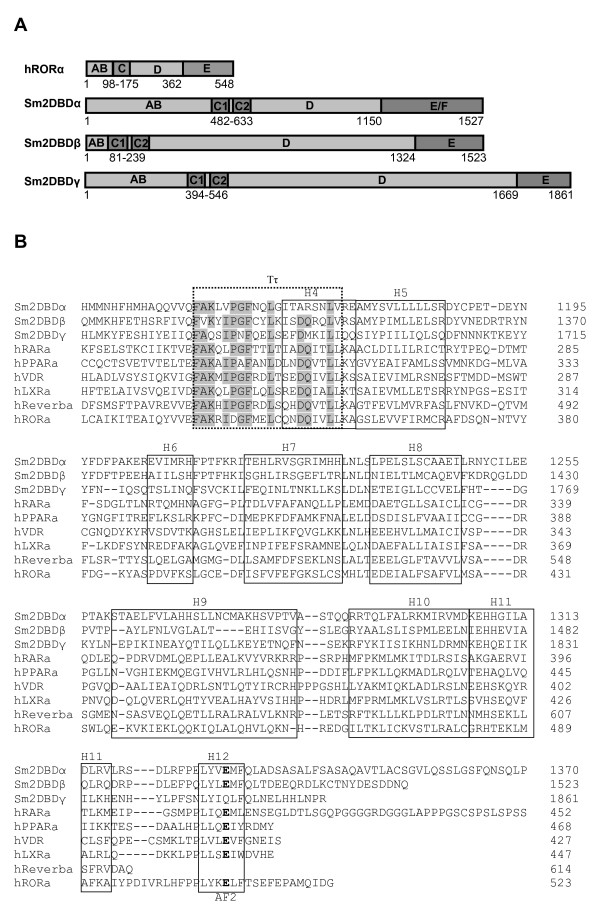
**Functional domains of 2DBD-Nuclear receptors and sequence alignment of the LBD domain**. **A. **Schematic representation of functional domains of 2DBD-NRs isolated from the fluke *Schistosoma mansoni*. hRORα (human RAR-related orphan receptor, NM_002943) as an example that shows the 'typical' modular structure of nuclear receptors, which contain an A/B domain, a C domain (DNA binding domain), a D domain (hinge region) and an E domain (ligand binding domain). Three Sm2DBD NRs (Sm2DBDα, Sm2DBDβ and Sm2DBDγ) exhibit a novel modular structure with an AB domain, two tandem DNA binding domains (C1 and C2), a D domain and an E domain. Sm2DBDα possesses an F domain at the C-terminal end of the E domain. The size of each domain in amino acids is indicated. **B. **Alignment of sequences from Helices 3–12 of the LBD domain of three *S. mansoni *2DBD-NRs with that of members in NR subfamily I. Helices described in [64] are boxed, the signature sequence of the LBD (Tτ) is boxed with dash line. The autonomous activation domain (AF2-AD) is indicated and the conserved glutamic acids are shown in bold. Numbers at the end of each line indicate residue positions in the original sequence, amino acids of Sm2DBDα 1371–1527 and hRARα 453–462 are not shown in the alignment. Dark shaded areas show conserved residues in the signature sequence of the LBD. The accession numbers of the aligned human nuclear receptors can be found in additional file 2.

cDNAs of three *S. mansoni 2DBD-NRs *(*Sm2DBDα*, 5144 bp, *Sm2DBDβ*, 5525 bp and *Sm2DBDγ*, 6374 bp) were isolated. Each cDNA encodes a large protein: *Sm2DBDα*, 1527 amino acids; *Sm2DBDβ*, 1523 amino acids and *Sm2DBDγ*, 1816 amino acids (Fig. 1A). Each protein exhibits a modular structure characteristic of the nuclear receptor superfamily with a divergent N terminal A/B domain, a hinge region and a less well conserved LBD. Remarkably, each possesses two DBDs in tandem (Fig. 1A). Thus the A/B-DBD-DBD-hinge-LBD organization represents a novel NR modular structure. All the members of this novel group have been placed in a new subfamily, NR VII.

The LBD is conserved in all three proteins from helix 3 to helix 12. The consensus signature sequence of the LBD (Tτ), ((F, WY)(A, SI)(K, R, E, G)XXX(F, L)XX(L, V, IXXX(D, S)(Q, K)XX(L, V)(L, I, F)) [1,4], is conserved in each of them (Fig. 1B). A putative AF2 activating domain core (AF2-AD), designated ΦΦXEΦΦ, where Φ represents a hydrophobic amino acid [13-15], is highly conserved in Sm2DBDα and Sm2DBDβ, but not in Sm2DBDγ. In Sm2DBDγ, a glutamine is located in the position which is normally conserved for a glutamic acid (Fig. 1B). Sm2DBDα contains a large F domain. The function of the F domain, which is known to be present in some but not all nuclear receptors, is not well known (eg. [15-22]). The hinge region of each protein is unusually large (Fig. 1A). This trait has been observed in other *Schistosoma *NRs [15-22]. The role of such a large hinge region is yet to be determined.

### Identification of *2DBD-NR *in other organisms

**Figure 2:**
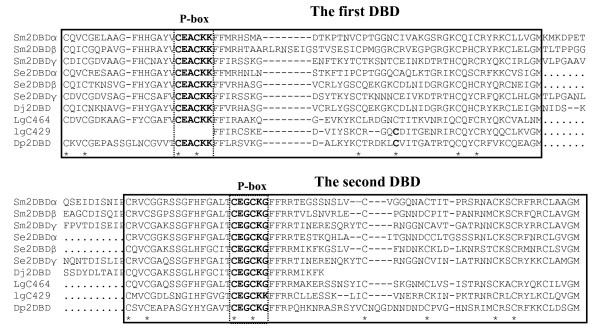
**Alignment of the deduced peptide sequences of DNA binding domains of 2DBD-NRs**. Each DBD is boxed with a solid line, P-box sequences (bold letters) are boxed with a dashed line. Stars identify the conserved cysteine residues that comprise the zinc finger of each DBD. Dashes indicate gaps in the sequence. The deduced amino acid sequence between DBDs for the NR from species other than *S. mansoni *is indicated with dots as we could not differentiate in the *in silico *analysis, exon sequence from intron sequence. All 2DBDs possess a P-box sequence of CEACKK in the first DBD and a P-box sequence of CEGCKG in the second DBD. Sm: flatworm *Schistosoma mansoni*, Se: flatworm *Schmidtea mediterranea*, Dj: flatworm *Dugesia japonica*, Lg: mollusk *Lottia gigantea*, Dp: arthropod *Daphnia pulex*.

**Figure 3:**
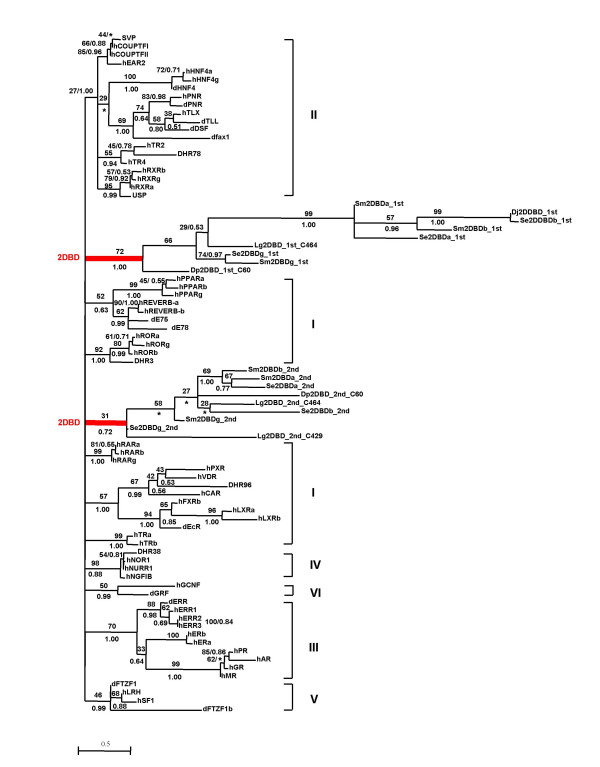
**Maximum Likelihood phylogenetic tree derived from sequences of DNA binding domains**. Amino acid sequences were aligned with ClustalW. Phylogenetic relationships were examined by the Maximum Likelihood (ML) method under Jones-Taylor-Thornton (JTT) substitution model with a gamma distribution of rates between sites (eight categories, parameter alpha, estimated by the program) using PHYML (v2.4.4)). Support values for the tree were obtained by bootstrapping a 100 replicates and are indicated above each branch. Branches under the threshold value of 27 (this value was set to support subfamily II as a monophyletic group) were shown as polytomies. The same data set was also tested by Bayesian inference. The trees were started randomly with four simultaneous Markov chains running for 5 million generations. Bayesian posterior probabilities (PPs) were calculated using a Markov chain Monte Carlo (MCMC) sampling approach implemented in MrBAYES v3.1.1, the PPs values are shown below each branch or after the ML bootstrapping value separated by a slash. Star indicates the node obtained form by Bayesian inference which was different from that obtained by ML method. The accession number of each sequence used for the phylogenetic analysis can be found in additional file 2 and 3.

By an extensive search of whole genomic sequence (WGS) databases extracted from NCBI, three *2DBD-NRs *were found in the freshwater turbellarian *Schmidtea mediterranea*, two were found in the mollusk *Lottia gigantean *(owl limpet) and one was found in the crustacean *Daphnia pulex *(water flea) (Fig. 2 and additional file 1). No *2DBD-NR *or any sequence encoding a P-box sequence of CEACKK, which is unique to the first DBD of 2DBD-NR (see below and Fig. 3), was found in sponge (*Reniera sp*), cnidarian (*Hydra magnipapillata and Nematostella vectensis*), arthropod insects, sea urchin or vertebrate sequence data bases. One additional *2DBD-NR *was found in the turbellarian *Dugesia japonica *in the NCBI EST database (Fig. 2 and additional file 1).

### Sequence analysis and phylogenetic tree construction

Alignment of the deduced DBD sequences showed that all 2DBD-NRs possess a P-box sequence, CEACKK, in the first DBD, and the P-box sequence, CEGCKG, in the second DBD (Fig. 2). A blast search against all available databases showed that the P-Box sequence CEACKK is not present in any other NR. This unique P-box present in the first DBD of 2DBD-NRs suggests a novel target DNA specificity may exist for the first DBD. The P-box sequence of second DBD, CEGCKG followed by the amino acid sequence FFRR (CEGCKGFFRR) is identical to that of most members in NR subfamily I (NR I) suggesting that 2DBD-NRs may have a close functional or evolutionary relationship with receptors in NR subfamily I.

**Figure 4:**
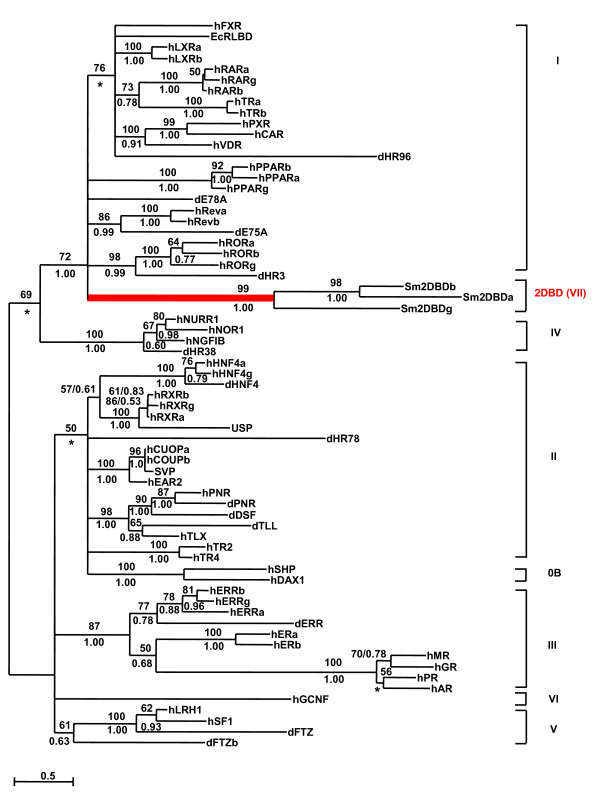
**Maximum Likelihood phylogenetic tree produced using sequences of NR ligand binding domain**. Phylogenetic relationship was examined by the Maximum Likelihood (ML) method as described for Fig. 3. Support values for the tree were obtained by bootstrapping a 100 replicates and are indicated above each branch. Branches under the threshold value of 46 (this value was set to support the subfamily IV as a monophyletic group) were shown as polytomies. Bayesian inference with the same methods as in Fig. 3, by running 3 million generations. The PPs are shown below each branch or after the ML bootstrapping value separated by a slash. Star indicates the node obtained form by Bayesian inference which was different from that obtained by the ML method. The accession number of each sequence used for phylogenetic analysis can be found in additiona1 file 2 and 3.

Both Maximum Likelihood method and Bayesian inference analysis show that the first DBD of 2DBD-NRs belongs to one monophyletic group and the second DBD belongs to a separate monophyletic group (Fig. 3). The results suggest that *2DBD-NRs *originated from a common ancestor gene. Both the first and the second DBD are sister groups to members of NR subfamily I, suggesting that the common ancestral gene of 2DBD-NRs was close to a common ancestral gene of NR subfamily I genes and the extra DBD was gained by a recombination event (Fig. 3). Another phylogenetic tree was constructed employing LBD sequences, the same result was obtained (Fig. 4). The mRNA sequences reported here were deposited in GenBank under the accession numbers: *Sm2DBDα *[GenBank:AY688250], *Sm2DBDβ *[GenBank:AY688251] and *Sm2DBDγ *[GenBank:AY698061]

### Protein-protein interaction

NRs can regulate transcription as a homodimer or as a heterodimer with retinoid × receptor (RXR), another nuclear receptor. To determine whether 2DBD-NRs may form dimers and to begin to define the quarternary structure of 2DBD-NRs, the interaction of Sm2DBDα, SmRXR1 and SmRXR2 was evaluated in a yeast two hybrid system.

**Figure 5:**
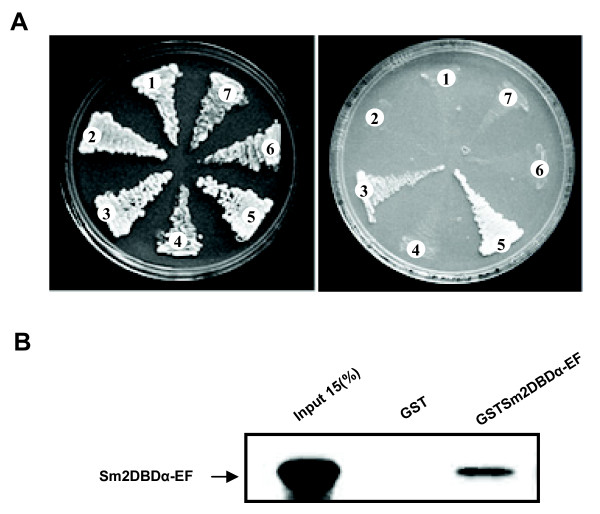
**Protein-protein interaction of Sm2DBDα**. **A. **Yeast two hybrid assays show that *S. mansoni *2DBD-NR (Sm2DBDα) can act as a homodimer but not as a heterodimer with SmRXR1 and SmRXR2. Yeast AH109 was transformed with 1 μg of the following co-transformats: 1) pGBK-Sm2DBDα-C-F/pACT-SmRXR1, 2) pGAD-Sm2DBDα/pAs-SmRXR1-C-F, 3) pGBK-Sm2DBDα-C-F/pGAD-Sm2DBDα, 4) negative control plasmid pSV40/plamin C, 5) positive control plasmid pSV40/p53, 6) pGBK-Sm2DBDα-C-F/pACT-SmRXR2, 7) pGAD-Sm2DBDα/pAS-SmRXR2. Transformed yeast were plated on SD/-trp-leu and SD/-trp-his-leu-ade plus 3 mM 3-amino-1,2,4-triazole (3-AT). The results show that Sm2DBDα can form a homodimer but not a heterodimer with SmRXRs. **B. **GST pull down verified that the *S. mansoni *2DBDα-E-F domain, in which the dimer interface is located, can form a homodimer *in vitro*.

Yeast transformed with pSV40/p53 (positive control), pSV40/plamin C (negative control), pGBK-Sm2DBDα-C-F/pACT-SmRXR1, pAS-SmRXR1-C-F/pGAD-Sm2DBDα, pGBK-Sm2DBDα-C-F/pACT-SmRXR2, pAS-SmRXR2/pGAD-Sm2DBDα and pGBK-Sm2DBDα-C-F/pGAD-Sm2DBDα grew on SD/-trp-leu plate as expected (Fig. 5A-a). If Sm2DBDα interacts with SmRXR1, SmRXR2 or with itself, the Gal4 binding domain fusion partner bound to the Gal1 UAS element will interact with the Gal4 activation domain to drive transcription of the reporter gene. Yeast co-transformed with pGBK-Sm2DBDα-C-F/pGAD-Sm2DBDα grew on SD/-trp-his-leu plus 3 mM 3-AT, indicating that Sm2DBDα can act as a homodimer. Yeast co-transformed with pGBK-Sm2DBDα-C-F/pACT-SmRXR1, pGBK-Sm2DBDα-C-F/pACT-SmRXR2 or pAS-SmRXR1-C-F/pGAD-Sm2DBDα and pAS-SmRXR2/pGAD-Sm2DBDα did not grow on SD/-trp-his-leu plus 3 mM 3-AT plates, indicating that Sm2DBDα did not interact with SmRXR1 nor SmRXR2 (Fig. 5A). The positive control plasmids, pSV40/p53, grew on SD/-trp-his-leu plus 3 mM 3-AT plates while the negative control plasmids, pSV40/plamin C did not (Fig. 5A). The results show that Sm2DBDα can interact as a homodimer, but can not interact with SmRXR1 nor SmRXR2. As a strong dimer interface is known to be located in the LBD [23,24], GST pull-down experiments were performed (Fig. 5B). The results verified that the LBDs of Sm2DBDα can form a homodimer *in vitro*.

Nuclear receptors act on target genes by recognizing and binding to specific DNA core motifs in the promoter region of target genes via their P-box motif located in the DBD. The DNA core motif is a typical consensus hexameric sequence AGGTCA called a half-site. When NRs bind to the half-site as a dimer, two P-boxes and a repeat of half-site, with different orientations and spacings between the half sites, are required. 2DBD-NR can interact as a homodimer indicating that four P-boxes may be involved in DNA binding, thus a novel mechanism of DNA binding, which requires two independent pairs of half-site repeats, or four half-site repeats, each with unknown orientations and spacing, are predicted to exist to allow 2DBD-NR to bind to target DNA cis-elements. The protein of the first DBD, second DBD and the first DBD with second DBD were tested for their ability to bind to a direct repeat, an everted repeat and palindromes of the half-site AGGTCA with 1–6 nucleotide spacings by electrphoretic mobility shift assay (EMSAs). No binding compared to controls was observed (data not shown). However, as the flanking sequence of the AGGTCA motif and the spacing between half sites also determines the protein binding to the DNA element, further experiments will be performed using different sets of templates and by determining DNA binding sites using a PCR/EMSA-based approach.

### Evolution of *2DBD-NRs*

**Figure 6:**
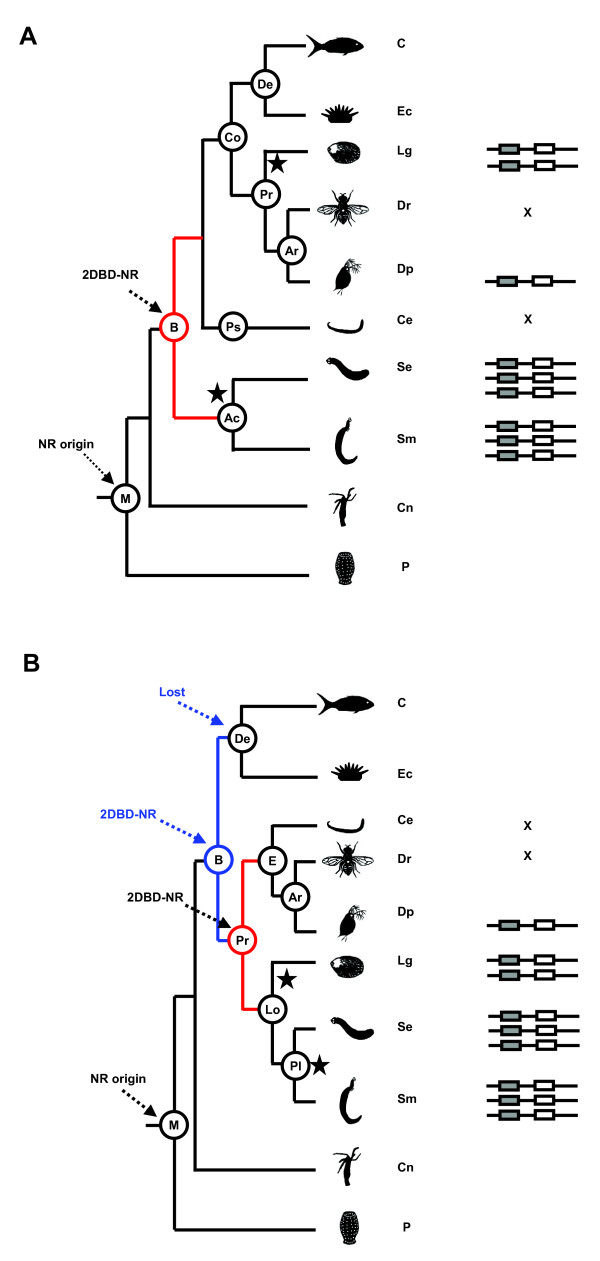
**Origin and duplication of *2DBD-NRs *in metazoans**. **A. **Deduction from traditional view of metazoan phylogeny. The common ancestor of *2DBD-NR *originated from DBD duplication in a common bilaterian ancestor (red branch). The fact that 2DBD-NRs are present in both Acoelomates and Coelomates supports this view. Star indicates duplication event(s). **B. **Deduction from the molecular view of metazoan phylogeny. The common ancestor of *2DBD-NR *might originate in a common Protostome ancestor (red branch). The fact that 2DBD-NRs are present in both Ecdysozoa and Lophotrochozoa supports this view. Another possibility is that *2DBD-NR *might originate in a common ancestor of the Bilateria and was lost in the Deuterostome lineage after the split of Protostomes and Deuterostomes (blue branch). Ac: Acoelomates, Ar: Arthropoda, B: Bilateria, C: Chordates, Ce: *Caenorhabditis elegans*, Cn: Cnidarians, Co: Coelomates, De: Deuterostomes, Dp: *Daphnia pulex*, Dr: Drosophila, E: Ecdysozoans, Ec: Echinoderms, Lg: *Lottia gigantean*, Lo: Lophotrochozoans, M: metazoa, Pl: Platyhelminths, Po: Poriferans, Pr: Protostomes, Ps: Pseudocoelomates, Se: *Schmidtea mediterranea*, Sm: *Schistosoma mansoni*, Ec: Echinoidea.  indicates 2DBD-NR, the number of  indicates the number of *2DBD-NRs *found in that taxon.

Metazoan phylogeny is still under debate [25-29]. In the traditional view based on morphological and embryological characteristics, Bilateria comprise Acoelomates (such as flatworms), Pseudocoelomates (Nematodes) and Coelomates (such as arthropods, mollusks and chordates) [25,27,29] (Fig. 6A). A second view based on molecular data (18S and 28S RNA genes, Hox genes, mitochondrial gene order, concatenated mitochondrial genes and myosin II heavy chain genes) support the Bilateria as comprising three clades: Deuterostomia, Lophotrochozoa and Ecdysozoa [26,28,30-33]. In the molecular phylogeny scheme, nematodes and arthropods are grouped into Ecdysozoa, while flatworms and mollusks are grouped into Lophotrochozoa (Fig. 6B). In an attempt to explain the origin of the ancestor *2DBD-NR*, both metazoan phylogenies were considered.

If the traditional phylogenetic scheme is correct, *2DBD-NRs *originated in a common ancestor of the Bilateria, because *2DBD-NRs *were found in both Acoelomates (flatworms) and Coelomates (mollusks and arthropods) (Fig. 6A). The *2DBD-NR *was lost in Pseudocoelomates (nematodes) after the split of Pseudocoelomates and Coelomates (Protostomes and Deuterostomes). As *2DBD-NRs *have not been found in Deuterostomes, they were lost in the Deuterostome lineage after the split of Protostomes and Deuterostomes (Fig. 6A).

If the molecular phylogeny hypothesis is correct, there were two possibilities for *2DBD-NR *origin. The *2DBD-NR *might originate in a common ancestor of Protostomes, since *2DBD-NRs *were identified both in Lophotrochozoans (Platyhelminths and Molluscs) and in Ecdysozoans (Crustaceans) (Fig. 6B). The other possibility is that *2DBD-NR *might originate in a common ancestor of the Bilateria and was lost in the Deuterostome lineage after the split of Protostomes and Deuterostomes. *2DBD-NR *is absent in nematodes suggesting that this gene was lost after the split of the nematodes and arthropods. In arthropods, no *2DBD-NR *was found in insects suggesting *2DBD-NR *was lost after the split of insects and crustaceans (Fig. 6B).

The phylogeny of the Platyhelminths has itself been under debate (eg. [34-37]). The Platyhelminths have always played a central role in hypotheses concerning metazoan phylogeny and evolution. Recently, many platyhelminth flatworms, previously regarded in the traditional phylogeny as basal bilaterians (Fig. 6A), are now placed within the lophotrochozoan protostomates (Fig. 6B). Furthermore, the Acoelomorpha (Aceola + Nerertoderdermatida) are no longer considered part of the Platyhelminths but are still considered basal bilaterians [34-38]. Certainly, further studies on nuclear receptor evolution in these taxa can contribute to our understanding of the evolution of the Metazoa and Bilateria, especially as nuclear receptors have been identified in sponges [39], a group that is hypothesized to have given rise to the hypothetical metazoan ancestor [40].

**Figure 7:**
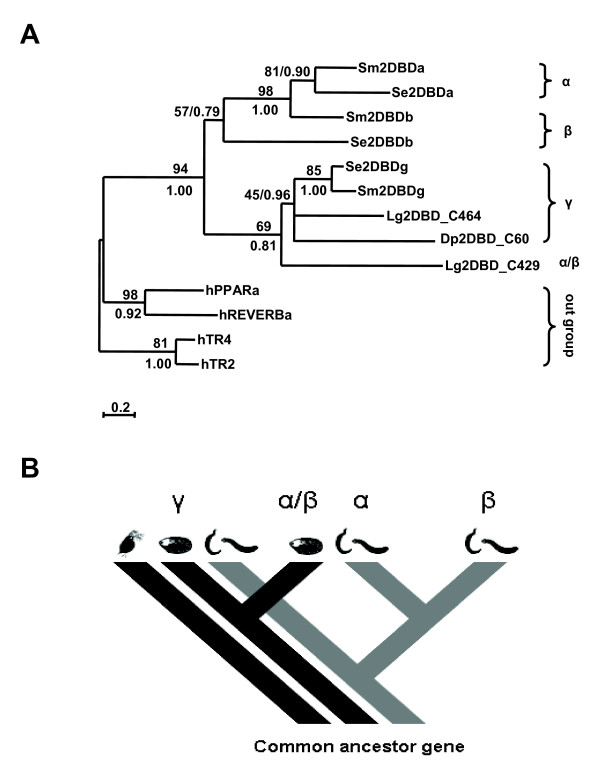
**Duplication of *2DBD-NRs***. **A. **Maximum Likelihood phylogenetic tree derived from sequences in the first and the second DBD of 2DBD-NRs. Phylogenetic relationship was examined by the Maximum Likelihood method as described for Fig. 3. Support values for the tree were obtained by bootstrapping a 100 replicates and are indicated above each branch. Branches under the threshold value of 45 were shown as polytomies. Bayesian inference with the same methods as in Fig. 3 running 5 million generations. The PPs are shown below each branch or after the ML bootstrapping value separated by a slash. **B. **Figure shows that the common ancestor gene of 2DBD-NRs underwent duplication after the split of the Arthropods, Molluscs and Platyhelminths. In Mollusks, the orthologue gene (γ) underwent a duplication giving rise to the α/β gene. In Platyhelminths, the orthologue gene (γ) underwent a duplication giving rise to a new gene, this new gene underwent a recent duplication to give birth to the two present genes (α and β genes). All three genes are present in the flatworm *S. mansoni *and the planarian *S. mediterranea *suggesting that the two rounds of 2DBD-NR duplication occurred in a common ancestor of the Platyhelminths.

The duplication of *2DBD-NRs *was deduced from phylogenetic analysis employing the first and the second DBD sequences as a unit. Maximum Likelihood method and Bayesian inference were performed and the same result was obtained (Fig. 7A). Since the results show that there is only one Arthropod, Mollusk and Platyhelminth *2DBD-NR *orthologue, the duplication of the ancient *2DBD-NR *first occurred after the split of Arthropods, Mollusks and Platyhelminths (Fig. 7B). The absence of a *2DBD-NR *in the insect and nematode lineages supports this hypothesis, since it is likely that there was less of a chance for all earlier duplicated genes to be lost in all the animals in which no *2DBD-NR *was found. The analysis also showed that *2DBD-NRs *underwent two rounds of duplication in a common ancestor of the Platyhelminths that eventually gave rise to three genes. The *2DBD-NRα *and *2DBD-NRβ *are considered the most recently duplicated, since the orthologue of *2DBD-NRγ *is found in Crustacea and Mollusca (Fig. 7A, B). Interestingly, a single gene is found in the crustacean, one duplication event occurred in mollusks and two duplications occurred in the Platyhelminths.

**Figure 8:**
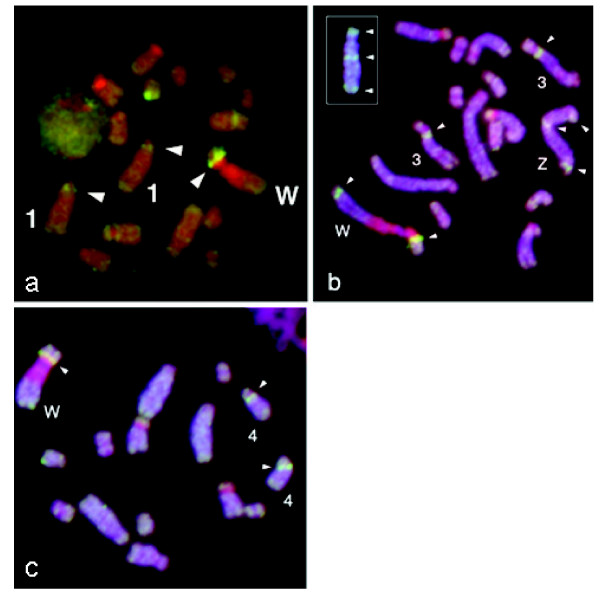
**Chromosome localization of *S. mansoni 2DBD-NRs***. *S. mansoni 2DBD-NRs *are located on different chromosomes as determined by FISH mapping using BAC DNA as a probe. **a. ***Sm2DBDα *(BAC: CHOR-18I10), **b. ***Sm2DBDβ *(BAC: SmBAC1 54O21), insert is the Z chromosome, **c. ***Sm2DBDγ *(BAC: SmBAC1 18F9). Chromosome numbers are indicated.

**Figure 9:**
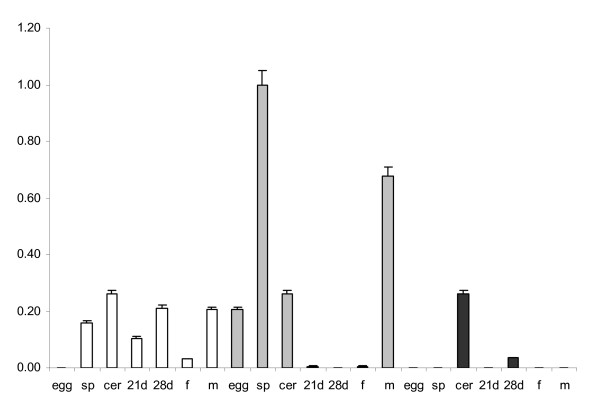
**Quantitative real-time RT-PCR shows mRNA expression of *S. mansoni 2DBD-NRs***. Normalized gene expression (ΔΔCT) of *Sm2DBDα, Sm2DBDβ *and *Sm2DBDγ *were standardized to the relative quantities of *S. mansoni *tubulin. For graphical representation of expression, the normalized expression was recalculated by dividing the expression level for each gene in each stage by the expression level of *Sm2DBDβ *from sporocysts, the highest expression level. egg: eggs, sp: daughter sporocysts, cer: cercariae, 21d: 21-day worms, 28d: 28-day worms, f: adult female worms and m: adult male worms.

*S. mansoni *bacterial artificial chromosome (BAC) clones containing *Sm2DBDα *(BAC: CHOR-18I10) were identified by screening the *S. mansoni *CHOR-1 BAC library. BAC clones containing *Sm2DBDβ *(BAC: SmBAC1 54O21) and *Sm2DBDγ *(BAC: SmBAC1 18F9) were identified by blast searching databases of *S. mansoni *BAC ends and verified by PCR. *Sm2DBDα, Sm2DBDβ *and *Sm2DBDγ *were localized on chromosomes by FISH using BAC DNA as the probe (Fig. 8). The results show that *Sm2DBDα *localized to chromosome 1. *Sm2DBDβ *is located on sex chromosomes Z and W and chromosome 3 indicating that there are two copies of *Sm2DBDβ*. However, there are other possibilities. It could be a fourth gene. However, we do not think that it is a fourth gene as we should have found it in our search of the genome sequence. It might be a pseudogene or a second copy of the *2DBDβ *gene. We favor a second copy of the genes as the probe hybridizes to the euchromatic region of the Z and W. However, we cannot rule out that it is an artifact due to hybridization of a repetitive sequence in the BAC clone. *Sm2DBDγ *is localized on chromosome 4. In figures 9a and 9c a repeat sequence in the BAC clones that commonly hybridizes to the W chromosome is shown as well. The results suggest that *2DBD-NRs *duplicated among different chromosomes in a common ancestor of the Platyhelminths.

Analysis of the NR superfamily, mainly in *Drosophila *and vertebrates supports the hypothesis that evolution of nuclear receptors occurred by two serial rounds of duplication [41-45]. The duplication of *2DBD-NRs *suggests that certain NR genes have undergone recent duplication in invertebrates after the divergence of various clades within the Bilateria. Our previous study of *S. mansoni *NRs supports this hypothesis [6]. NRs in insects seem to have undergone extensive gene loss. For example, a recently identified estrogen receptor in the mollusk *Aplysia californica *[46] and two thyroid hormone receptors in the Platyhelminth *S. mansoni *[6,47] are missing in the insect genera *Drosophila *and *Anopheles*. To address the importance of gene duplication in NR evolution, more invertebrates NR complements await to be analyzed.

### Developmentally Regulated Expression

Quantitative real-time RT-PCR was performed to evaluate mRNA expression of *Sm2DBDα, Sm2DBDβ *and *Sm2DBDγ*. Normalized gene expression (ΔΔCT) [48] was standardized to the relative quantities of *S. mansoni *α-tubulin. *Sm2DBDα *was detected in secondary sporocysts, cercariae, 21-day schistosomules, 28-day schistosomules, female and male worms. *Sm2DBDβ *was expressed relatively high in eggs, secondary sporocysts, cercariae and male stages. Sm2DBD*γ *was only detected in cercariae and 28-day worms. The results indicate that the three genes are developmentally regulated and thus have a role in different development stages (Fig. 9). It is of note that Sm2DBDγ, the putative ancestral gene is only expressed in 2 of the developmental stages studied and that both Sm2DBDα and Sm2DBDβ show sex-specific gene expression.

## Conclusion

A protein modular structure containing an AB domain, two DNA binding domains in tandem, a hinge region and a ligand binding domain (A/B-DBD-DBD-hinge-LBD) represents a novel NR modular structure, and is named 2DBD-NR. *2DBD-NR*s were identified from mollusks, arthropods (crustaceans) and flatworms. 2DBD-NRs may act as homodimers. *2DBD-NR*s share a common ancestor gene which possessed an extra DBD that likely resulted from a recombination event. *2DBD-NR*s were found in flatworms, mollusks and arthropods whose phylogeny is still under debate [30,31,33] (Fig. 6A and 6B). Further studies of *2DBD-NR *gene subfamily may contribute to our understanding of gene duplication as an evolutionary force and to the phylogeny of the Metazoa. The conserved zinc finger motifs in each of the two DBDs are the most readily recognized features of 2DBD-NRs. The P-box sequences in the first DBD and the second DBD give members of the 2DBD-NR their unique feature. This feature makes 2DBD-NRs an interesting gene subfamily for studies of metazoan phylogeny.

## Methods

### Isolation of 2DBD-NR cDNAs in the Platyhelminth *S. mansoni*

cDNAs encoding the entire open reading frame (ORF) of three *S. mansoni *2DBD-NRs (Sm2DBDα, Sm2DBDβ and Sm2DBDγ) were isolated by a PCR strategy using a *S. mansoni *female worm cDNA library (pBluescript SK (+/-) phagemid) pool as template DNA [6,22]. The PCR primers for one end (either 5' or 3' end) were designed according to a fragment encoding the previously identified DBD region of these genes [6]. The primer for the other end (either 5' or 3' end) was a vector universal primer (M13-Rev and T3, or M13-For and T7 primers). PCR products were separated on 1.2% agarose gels, ligated into pCR2.1 TOPO vector (Invitrogen) and sequenced. After the correct fragments were identified, the cDNA sequence containing the 5' UTR, ORF and 3' UTR were obtained by PCR. The cDNAs were shown to be related to a single mRNA species by sequencing the PCR products obtained from single-stranded cDNA using primers located within the 5'UTR and 3'UTR of each gene.

### Data mining

Whole genomic sequences (WGS) were extracted from the GenBank public ftp site [49] (up to October 2005) and imported into StarBlast program (DNASTAR) to build a local database which was screened by tblastn using the sequence of the first and second DBD of Sm2DBDα as the query. Any sequence that contained a zinc finger structure of the DBD (Cys-X2-Cys-X13-Cys-X2-Cys or Cys-X5-Cys-X9-Cys-X2-Cys) was retained. Sequence walking was carried out to assemble the contigs. Website databases of GenBank (nr, EST_human, EST_mouse and EST_other databases) [50], European Bioinformatics Institute [51] and Swiss-Prot [52] were also mined by tblasn or blastp using the same query sequence as above.

### Phylogenetic tree construction

Phylogenetic trees were constructed from deduced sequences of the DBD and the LBD, respectively. Sequences were aligned with ClustalW [53]. Phylogenetic analysis of the data set was carried out using the Maximum Likelihood method under Jones-Taylor-Thornton (JTT) substitution model [54] with a gamma distribution of rates between sites (eight categories, parameter alpha, estimated by the program) using PHYML (v2.4.4)) [55]. Support values for the tree were obtained by bootstrapping a 100 replicates. The same data set was also tested by Bayesian inference using MrBAYES v3.1.1 with a mixed amino acid replacement model + invgamma rates (Huelsenbeck and Ronquist, 2001). The trees were started randomly; four simultaneous Markov chains were run for 5 million generations for the DBD data set and 3 million generations for the LBD data set, respectively. The trees were sampled every 100 generations. Bayesian posterior probabilities (PPs) were calculated using a Markov chain Monte Carlo (MCMC) sampling approach implemented in MrBAYES v3.1.1, with a burn-in value setting at 12,500 for DBD data set and 7,500 generations for the LBD data set, respectively.

### Protein-protein interaction

Yeast two-hybrid Assay: cDNA encoding Sm2DBDα was inserted into the activation domain vector pGAD-T7 to form pGAD-Sm2DBDα. Since Sm2DBDα-AB domain can self-activate as previously determined (data not shown), cDNA encoding Sm2DBDα C-F domain was inserted into pGBK-T7 to form pGBK-Sm2DBDα-C-F. Previously constructed SmRXR1, SmRXR1-C-F and SmRXR2 in activation domain vectors (pACT-SmRXR1 and pACT-SmRXR2), and in DNA binding domain vectors (pAS-SmRXR1-CF and pAS-SmRXR2-AF) were employed [17,18,56,57]. Yeast AH109 were transformed with 1 μg of the following co-transformants: pGBK-Sm2DBDα-C-F/pACT-SmRXR1, pGAD-Sm2DBDα/pAs-SmRXR1-C-F, pGBK-Sm2DBDα-C-F/pACT-SmRXR2, pGAD-Sm2DBDα/pAS-SmRXR2, pGBK-Sm2DBDα-C-F/pGAD-Sm2DBDα, positive control plasmid pSV40/p53 and negative control plasmid pSV40/plamin C. Transformations were performed using the Frozen-EZ transformation II kit (Zymo Research). Transformed yeast were plated on SD/-trp-leu and SD/-trp-his-leu-ade plus 3 mM 3-amino-1,2,4-triazole (3-AT, an inhibitor to prevent the leaky expression of the HIS3 gene).

GST Pull-down: cDNA encoding Sm2DBDα E-F domain was inserted into pGEX-4T-1 and pCITE-4a vectors to form pGEX-Sm2DBDα-E-F and pCITE-Sm2DBDα-E-F, respectively. *E. coli *AD 494 (DE3) pLys S competent cells (Novagen) were transformed with pGEX-Sm2DBDα-E-F and the GST fusion proteins were purified by passage over a glutathione-Sepharose column according to standard protocols. To produce ^35^S labeled protein, pCITE-Sm2DBDα-E-F was transcribed and translated using the Single Tube Protein System (Novagen) following the manufacture's protocol. For pull-down experiments, a 50 μl reaction that contained 2 μl of the *in vitro *translation reaction, Sm2DBDα-E-F GST fusion protein or GST protein (as negative control) affixed to glutathione-Sepharose beads (about 2 μg) and binding buffer (50 mM Tris-HCl, pH 7.5, 100 mM NaCl, 10% glycerol, 0.15% Nonidet P40) was used [58]. The reaction was incubated overnight at 4°C, washed three times with binding buffer and the bound proteins were analyzed by 10% SDS-PAGE and autoradiography.

### BAC clone screening and localization of *2DBD-NRs *on chromosomes of *S. mansoni*

*S. mansoni *BAC clones containing *Sm2DBD-NRs *were identified by screening the *S. mansoni *CHOR-1 BAC library with methods previously described [59] or by blast searching databases of *S. mansoni *BAC end sequences in TIGR [60] and verified by PCR. Fluorescent *in situ *hybridization (FISH) was performed on *S. mansoni *sporocyst metaphase chromosome spreads with BAC DNAs that each contained one of the three *S. mansoni 2DBD-NRs *(*Sm2DBDα, Sm2DBDβ and Sm2DBDγ*). FISH was performed using techniques previously described [61,62].

### Quantitative real-time RT-PCR

mRNA expression levels of three *Sm2DBD-NRs *(*Sm2DBDα*, *Sm2DBDβ *and *Sm2DBDγ*) were tested in eggs, daughter sporocysts, cercariae, 21-day, 28-day, adult female and adult male worms. A Puerto-Rican strain of *S. mansoni *was maintained in snails(*Biomphalaria glabrata*) and Syrian golden hamsters (*Mesocricetus auratus*). Cercariae were released from infected snails and harvested on ice. Schistosome worms of different ages (21–45 day-old) were harvested from infected Syrian golden hamsters. Single-sex worms were obtained by separating adult worm pairs. Parasite eggs were obtained from livers of infected hamsters. Total RNA was extracted from the above developmental stages using TRIzol reagent (Invitrogen). All RNA samples were treated with RNase-free DNaseI (RQ1 DNase; Promega) and reverse transcribed using a random hexamer and SuperScript Reverse Transcriptase II (SSRTaseII; Invitrogen) as previously described [6]. Primers specific for *Sm2DBDα *(forward: 5'-CCGCTGCATCAATCACCTATT-3', reverse: 5'-TGCGCAAAATGTAGCCGAT-3'), *Sm2DBDβ *(forward: 5'-TGCACTGACTCCCACCACA-3', reverse: 5'-AGCAGTGGATGACGTCGGA-3')*and Sm2DBDγ *(forward: 5'-GAACATCGTGAATCAATTTTACATTCAG-3', reverse: 5'- ATGTACTGTTTCATTGCATTCATTTG-3') were designed using Primer Express Program (Applied Biosystems™). Primers specific for *S. mansoni *α-tubulin [GenBank: M80214] were according to [63]. Reverse-transcribed cDNA samples were used as templates for PCR amplification using SYBR Green Master Mix^® ^(Invitrogen) and BIORAD IQ™5 Real-Time PCR Detection System. The efficiency for each primer set is evaluated and recorded during assay development by iQ5 application (cDNA diluted to x1, x10, x100 and x1000 folds). Normalized gene expression (ΔΔCT) [48] of *Sm2DBDα, Sm2DBDβ *and *Sm2DBDγ *were standardized to the relative quantities of *S. mansoni *tubulin using BioRad IQ™5 Optical System software V1.1 with the Normalized Expression calculations implemented in iQ5. For graphical representation of the expression, the normalized expression was recalculated by dividing the expression level of each stage of the all gene by the highest expression level.

## List of abbreviations

2DBD-NR: nuclear receptor containing two tandem DNA binding domains, BAC: bacterial artificial chromosome, DBD: DNA-binding domain, FISH: Fluorescent *in situ *hybridization, LBD: ligand binding domain, NR: nuclear receptor, WGS: whole genomic sequence.

## Authors' contributions

WW performed the experiments and analysis. He is the primary author of the manuscript.

EGN was responsible for the design and analysis of the experiments. He contributed to the writing of the manuscript.

HH performed the FISH experiments and contributed to the writing of the manuscript.

PTL was responsible for the overall design, analysis and interpretation of the results. He contributed to the writing and preparation of the manuscript.

## Supplementary Material

Additional File 1 lists of genomic or EST sequences encoding 2DBD-NR identified by data mining Click here for file

Additional File 2 lists of GenBank accession number of cDNA of human NRs analyzed in this study Click here for file

Additional File 3 lists of GenBank accession number of cDNA sequences of *D. melanogaster *NRs analyzed in this study Click here for file

## References

[B1] Wurtz JM, Bourguet W, Renaud JP, Vivat V, Chambon P, Moras D, Gronemeyer H (1996). A canonical structure for the ligand-binding domain of nuclear receptors. Nat Struct Biol.

[B2] Renaud JP, Moras D (2000). Structural studies on nuclear receptors. Cell Mol Life Sci.

[B3] de Groot A, de Rosny E, Juillan-Binard C, Ferrer JL, Laudet V, Pierce RJ, Pebay-Peyroula E, Fontecilla-Camps JC, Borel F (2005). Crystal structure of a novel tetrameric complex of agonist-bound ligand-binding domain of Biomphalaria glabrata retinoid X receptor. J Mol Biol.

[B4] Wang LH, Tsai SY, Cook RG, Beattie WG, Tsai MJ, O'Malley BW (1989). COUP transcription factor is a member of the steroid receptor superfamily. Nature.

[B5] Moras D, Gronemeyer H (1998). The nuclear receptor ligand-binding domain: structure and function. Curr Opin Cell Biol.

[B6] Wu WJ, Niles EG, El-Sayed N, Berriman M, LoVerde PT (2006). Schistosoma mansoni (Platyhelminthes, Trematoda) nuclear receptors: Sixteen new members and a novel subfamily. Gene.

[B7] Nauber U, Pankratz MJ, Kienlin A, Seifert E, Klemm U, Jackle H (1988). Abdominal segmentation of the Drosophila embryo requires a hormone receptor-like protein encoded by the gap gene knirps. Nature.

[B8] Oro AE, Ong ES, Margolis JS, Posakony JW, McKeown M, Evans RM (1988). The Drosophila gene knirps-related is a member of the steroid-receptor gene superfamily. Nature.

[B9] Rothe M, Nauber U, Jackle H (1989). Three hormone receptor-like Drosophila genes encode an identical DNA-binding finger. Embo J.

[B10] Sengupta P, Colbert HA, Bargmann CI (1994). The C. elegans gene odr-7 encodes an olfactory-specific member of the nuclear receptor superfamily. Cell.

[B11] Seol W, Choi HS, Moore DD (1996). An orphan nuclear hormone receptor that lacks a DNA binding domain and heterodimerizes with other receptors. Science.

[B12] Zanaria E, Muscatelli F, Bardoni B, Strom TM, Guioli S, Guo W, Lalli E, Moser C, Walker AP, McCabe ER (1994). An unusual member of the nuclear hormone receptor superfamily responsible for X-linked adrenal hypoplasia congenita. Nature.

[B13] Nagy L, Kao HY, Love JD, Li C, Banayo E, Gooch JT, Krishna V, Chatterjee K, Evans RM, Schwabe JW (1999). Mechanism of corepressor binding and release from nuclear hormone receptors. Genes Dev.

[B14] Perissi V, Staszewski LM, McInerney EM, Kurokawa R, Krones A, Rose DW, Lambert MH, Milburn MV, Glass CK, Rosenfeld MG (1999). Molecular determinants of nuclear receptor-corepressor interaction. Genes Dev.

[B15] de Mendonca RL, Escriva H, Bouton D, Zelus D, Vanacker JM, Bonnelye E, Cornette J, Pierce RJ, Laudet V (2000). Structural and functional divergence of a nuclear receptor of the RXR family from the trematode parasite Schistosoma mansoni. Eur J Biochem.

[B16] de Mendonca RL, Bouton D, Bertin B, Escriva H, Noel C, Vanacker JM, Cornette J, Laudet V, Pierce RJ (2002). A functionally conserved member of the FTZ-F1 nuclear receptor family from Schistosoma mansoni. Eur J Biochem.

[B17] Freebern WJ, Niles EG, LoVerde PT (1999). RXR-2, a member of the retinoid x receptor family in Schistosoma mansoni. Gene.

[B18] Freebern WJ, Osman A, Niles EG, Christen L, LoVerde PT (1999). Identification of a cDNA encoding a retinoid X receptor homologue from Schistosoma mansoni. Evidence for a role in female-specific gene expression. J Biol Chem.

[B19] Hu R, Wu W, Niles EG, LoVerde PT (2006). Isolation and characterization of Schistosoma mansoni constitutive androstane receptor. Molecular and Biochemical Parasitology.

[B20] Hu R, Wu W, Niles EG, Loverde PT (2006). SmTR2/4, a Schistosoma mansoni homologue of TR2/TR4 orphan nuclear receptor. Int J Parasitol.

[B21] Lu C, Wu W, Niles EG, Loverde PT (2006). Identification and characterization of a novel fushi tarazu factor 1 (FTZ-F1) nuclear receptor in Schistosoma mansoni. Mol Biochem Parasitol.

[B22] Wu W, Niles EG, Hirai H, Loverde PT (2007). Identification and characterization of a nuclear receptor subfamily I member in the Platyhelminth Schistosoma mansoni (SmNR1). Febs J.

[B23] Bourguet W, Vivat V, Wurtz JM, Chambon P, Gronemeyer H, Moras D (2000). Crystal structure of a heterodimeric complex of RAR and RXR ligand-binding domains. Mol Cell.

[B24] Gampe RT, Montana VG, Lambert MH, Miller AB, Bledsoe RK, Milburn MV, Kliewer SA, Willson TM, Xu HE (2000). Asymmetry in the PPARgamma/RXRalpha crystal structure reveals the molecular basis of heterodimerization among nuclear receptors. Mol Cell.

[B25] Blair JE, Ikeo K, Gojobori T, Hedges SB (2002). The evolutionary position of nematodes. BMC evolutionary biology.

[B26] Copley RR, Aloy P, Russell RB, Telford MJ (2004). Systematic searches for molecular synapomorphies in model metazoan genomes give some support for Ecdysozoa after accounting for the idiosyncrasies of Caenorhabditis elegans. Evolution & development.

[B27] Dopazo H, Santoyo J, Dopazo J (2004). Phylogenomics and the number of characters required for obtaining an accurate phylogeny of eukaryote model species. Bioinformatics (Oxford, England).

[B28] Philippe H, Lartillot N, Brinkmann H (2005). Multigene analyses of bilaterian animals corroborate the monophyly of Ecdysozoa, Lophotrochozoa, and Protostomia. Mol Biol Evol.

[B29] Wolf YI, Rogozin IB, Koonin EV (2004). Coelomata and not Ecdysozoa: evidence from genome-wide phylogenetic analysis. Genome Res.

[B30] Aguinaldo AM, Turbeville JM, Linford LS, Rivera MC, Garey JR, Raff RA, Lake JA (1997). Evidence for a clade of nematodes, arthropods and other moulting animals. Nature.

[B31] Halanych KM, Bacheller JD, Aguinaldo AM, Liva SM, Hillis DM, Lake JA (1995). Evidence from 18S ribosomal DNA that the lophophorates are protostome animals. Science.

[B32] Halanych KM (2004). The new view of animal phylogeny. Annu Rev Ecol Evol Syst.

[B33] Winnepenninckx B, Backeljau T, Mackey LY, Brooks JM, De Wachter R, Kumar S, Garey JR (1995). 18S rRNA data indicate that Aschelminthes are polyphyletic in origin and consist of at least three distinct clades. Mol Biol Evol.

[B34] Baguna J, Riutort M (2004). The dawn of bilaterian animals: the case of acoelomorph flatworms. Bioessays.

[B35] Baguna J, Riutort M (2004). Molecular phylogeny of the Platyhelminthes. Can J Zool.

[B36] Jondelius U, Ruiz-Trillo I, Baguna J, Riutort M (2002). The Nemertodermatida are basal bilaterians and not members of the Platyhelminthes. Zoologica Scripta.

[B37] Telford MJ, Lockyer AE, Cartwright-Finch C, Littlewood DT (2003). Combined large and small subunit ribosomal RNA phylogenies support a basal position of the acoelomorph flatworms. Proceedings.

[B38] Ruiz-Trillo I, Paps J, Loukota M, Ribera C, Jondelius U, Baguna J, Riutort M (2002). A phylogenetic analysis of myosin heavy chain type II sequences corroborates that Acoela and Nemertodermatida are basal bilaterians. Proc Natl Acad Sci U S A.

[B39] Wiens M, Batel R, Korzhev M, Muller WE (2003). Retinoid X receptor and retinoic acid response in the marine sponge Suberites domuncula. J Exp Biol.

[B40] Muller WE, Schroder HC, Skorokhod A, Bunz C, Muller IM, Grebenjuk VA (2001). Contribution of sponge genes to unravel the genome of the hypothetical ancestor of Metazoa (Urmetazoa). Gene.

[B41] Bertrand S, Brunet FG, Escriva H, Parmentier G, Laudet V, Robinson-Rechavi M (2004). Evolutionary genomics of nuclear receptors: from twenty-five ancestral genes to derived endocrine systems. Mol Biol Evol.

[B42] Escriva H, Bertrand S, Laudet V (2004). The evolution of the nuclear receptor superfamily. Essays Biochem.

[B43] Escriva H, Langlois MC, Mendonca RL, Pierce R, Laudet V (1998). Evolution and diversification of the nuclear receptor superfamily. Ann N Y Acad Sci.

[B44] Laudet V, Hanni C, Coll J, Catzeflis F, Stehelin D (1992). Evolution of the nuclear receptor gene superfamily. Embo J.

[B45] Thornton JW (2003). Nonmammalian nuclear recptors: Evolution and endocrine disruption. Pure Appl Chem.

[B46] Thornton JW, Need E, Crews D (2003). Resurrecting the ancestral steroid receptor: ancient origin of estrogen signaling. Science.

[B47] Verjovski-Almeida S, DeMarco R, Martins EA, Guimaraes PE, Ojopi EP, Paquola AC, Piazza JP, Nishiyama MY, Kitajima JP, Adamson RE, Ashton PD, Bonaldo MF, Coulson PS, Dillon GP, Farias LP, Gregorio SP, Ho PL, Leite RA, Malaquias LC, Marques RC, Miyasato PA, Nascimento AL, Ohlweiler FP, Reis EM, Ribeiro MA, Sa RG, Stukart GC, Soares MB, Gargioni C, Kawano T, Rodrigues V, Madeira AM, Wilson RA, Menck CF, Setubal JC, Leite LC, Dias-Neto E (2003). Transcriptome analysis of the acoelomate human parasite Schistosoma mansoni. Nat Genet.

[B48] Livak KJ, Schmittgen TD (2001). Analysis of relative gene expression data using real-time quantitative PCR and the 2(-Delta Delta C(T)) Method. Methods.

[B49] GenBank public ftp site. ftp://ftp.ncbi.nlm.nih.gov/pub/TraceDB/.

[B50] GenBank. http://www.ncbi.nlm.nih.gov/BLAST/.

[B51] European Bioinformatics Institute. http://www.ebi.ac.uk/blast2.

[B52] Swiss-Prot. http://www.expasy.ch/sprot/.

[B53] ClustalW. http://www.cf.ac.uk/biosi/research/biosoft/Downloads/clustalw.html.

[B54] Jones DT, Taylor WR, Thornton JM (1992). The rapid generation of mutation data matrices from protein sequences. Comput Appl Biosci.

[B55] Guindon S, Gascuel O (2003). A simple, fast, and accurate algorithm to estimate large phylogenies by maximum likelihood. Syst Biol.

[B56] Fantappie MR, Freebern WJ, Osman A, LaDuca J, Niles EG, LoVerde PT (2001). Evaluation of Schistosoma mansoni retinoid X receptor (SmRXR1 and SmRXR2) activity and tissue distribution. Mol Biochem Parasitol.

[B57] Fantappie MR, Osman A, Ericsson C, Niles EG, LoVerde PT (2003). Cloning of Schistosoma mansoni Seven in Absentia (SmSINA)(+) homologue cDNA, a gene involved in ubiquitination of SmRXR1 and SmRXR2. Mol Biochem Parasitol.

[B58] Osman A, Niles EG, LoVerde PT (2001). Identification and characterization of a Smad2 homologue from Schistosoma mansoni, a transforming growth factor-beta signal transducer. J Biol Chem.

[B59] Le Paslier MC, Pierce RJ, Merlin F, Hirai H, Wu W, Williams DL, Johnston D, LoVerde PT, Le Paslier D (2000). Construction and characterization of a Schistosoma mansoni bacterial artificial chromosome library. Genomics.

[B60] TIGR. http://tigrblast.tigr.org/er-blast/index.cgi?project=sma1.

[B61] Hirai H, LoVerde PT (1995). FISH techniques for constructing physical maps on schistosome chromosomes. Parasitol Today.

[B62] Hirai H, Hirai Y (2004). FISH mapping for helminth genome. Methods Mol Biol.

[B63] Oger F, Bertin B, Caby S, Dalia-Cornette J, Adams M, Vicogne J, Capron M, Pierce RJ (2006). Molecular cloning and characterization of Schistosoma mansoni Ftz-F1 interacting protein-1 (SmFIP-1), a novel corepressor of the nuclear receptor SmFtz-F1. Mol Biochem Parasitol.

[B64] Renaud JP, Rochel N, Ruff M, Vivat V, Chambon P, Gronemeyer H, Moras D (1995). Crystal structure of the RAR-gamma ligand-binding domain bound to all-trans retinoic acid. Nature.

