# Supplementary material for: Evolution of a novel subfamily of nuclear receptors with members that each contain two DNA binding domains
Source: BMC Evol Biol. 2007 Feb 23;7:27. doi: 10.1186/1471-2148-7-27 (PMC1810520; doi:10.1186/1471-2148-7-27)
Supplement: Additional File 1 — lists of genomic or EST sequences encoding 2DBD-NR identified by data mining [file 1471-2148-7-27-S1.doc]

Additional file 1. **ID of genomic or EST sequences encoding 2DBD-NR identified by data mining**

| Animals | GenBank gi (name of sequence) |
| --- | --- |
| *Schmidtea mediter* | 2DBDα:  gnl|ti|314350949 (uey24f11.g1), gnl|ti|513938606 (SAAA-ack82d03.b1), gnl|ti|529409931 (SAAA-aih12d09.g1), gnl|ti|511924710 (SAAA-aag12d01.g1), gnl|ti|518667560 (SAAA-acx75f08.g1), gnl|ti|561653701 (SAAA-acx75f08.g3), gnl|ti|564720257 (SAAA-apr23c01.g1), gnl|ti|516922935 (SAAA-abh34g07.g1), gnl|ti|517251253 (SAAA-acc71a08.g1), gnl|ti|313960310 (udp40c01.b1), gnl|ti|314408568 (ufd91a08.b1), gnl|ti|536855680 (SAAA-ald24d12.g1), gnl|ti|574897754 (SAAA-asc71g01.b1)  2DBDβ:  gnl|ti|543506943 (SAAA-amz14g08.g1), gnl|ti|523333014 (SAAA-aga69f10.g1), gnl|ti|527464783 (SAAA-aid45d09.g1), gnl|ti|574414957 (SAAA-arf71b08.b1), gnl|ti|526759310 (SAAA-aik37d03.b1), gnl|ti|536027614 (SAAA-alh05g01.g1)  2DBDγ:  gnl|ti|543159404 (SAAA-anb43a01.b1), gnl|ti|317045555 (ujj80b08.b1), gnl|ti|512898198 (SAAA-abv49f01.b1), gnl|ti|550357107 (SAAA-amd67g05.g1), gnl|ti|543766313 (SAAA-amz00g09.g1), gnl|ti|573614908 (SAAA-apz20e06.g1), gnl|ti|524211483 (SAAA-aib61g06.g1), gnl|ti|314100661 (ueb75c10.g1), gnl|ti|529818436 (SAAA-ajz55g04.b1), gnl|ti|314518090 (ufw39f06.b1), gnl|ti|573543718 (SAAA-aqe49c09.g1), gnl|ti|574446469 (SAAA-arq29d10.g1), gnl|ti|527457828 (SAAA-ail44a03.g1), gnl|ti|544353851 (SAAA-amz00g09.b1) |
| *Dugesia japonica* | BP186725 |
| *Lottia gigantea* | C464 (2DBDγ:):  gnl|ti|827939032 (AZYG246453.b1), gnl|ti|828173158 (AZYG305707.b2), gnl|ti|828261463 (AZYG323452.g1), gnl|ti|828297008 (AZYG334230.b1), gnl|ti|828491162, (AZYG383264.b1), gnl|ti|828806600 (AZYG509059.b1), gnl|ti|829164036 (AZZI144315.y1), gnl|ti|829170932 (AZZI144315.x1), gnl|ti|829242189 (AZZI174807.y1), gnl|ti|829336659 (AZYH26054.g1), gnl|ti|829554982 (AZYH90691.b1), gnl|ti|829572444 (AZZI264849.x1), gnl|ti|829572826 (AZZI264849.y1), gnl|ti|829705767 (AZZI291239.x1), gnl|ti|829999289 (AZZI381227.y1), gnl|ti|833350195 (AZZI467424.y1), gnl|ti|838062726 (AZZI498169.x1), gnl|ti|844115613 (AZYG131211.g1), gnl|ti|844432317 (AZZI599232.x1), gnl|ti|844432701 (AZZI599232.y1), gnl|ti|844552978 (AZZI724119.y1), gnl|ti|845901675 (AZYG864373.g1),gnl|ti|846311739 (AZZI633403.g1), gnl|ti|848863844 (AZZI836368.x1), gnl|ti|850070700 (AZZI845359.y1), gnl|ti|850075800 (AZZI848560.y1), gnl|ti|850627958 (AZZI933635.x1), gnl|ti|855139071 (AZZI926945.x1), gnl|ti|855143292 (AZZI926945.y1), gnl|ti|946489707 (BGNG33633.b1), gnl|ti|953133400 (BGNG147408.g1), gnl|ti|956739735 (BGOB237150.b1), gnl|ti|963828589 (BGOB182673.b1), gnl|ti|964790300 (BGNG229506.g1), gnl|ti|964795673 (BGNG233439.b1), gnl|ti|976495683 (BGNG245009.b1), gnl|ti|976496067 (BGNG245009.g1)  C429 (2DBDα/β:):  gnl|ti|829909115 (AZZI341340.y1), gnl|ti|828329628 (AZYG341890.g1), gnl|ti|828682703 (AZYG482821.b1), gnl|ti|836380590 (AZYG737582.g1), gnl|ti|828904158 (AZYG545395.g1), gnl|ti|828704936 (AZZI4651.y1), gnl|ti|828483115 (AZYG378961.g1), gnl|ti|835382848 (AZYG728736.b1), gnl|ti|828824016 (AZYG523528.g1), gnl|ti|827479073 (AZYG149917.b1), gnl|ti|836593018 (AZZI473235.x1), gnl|ti|829684529 (AZYH133843.b3), gnl|ti|830069581 (AZZI439487.x1), gnl|ti|827003956 (AZYG66780.g1), gnl|ti|836416724 (AZYG803860.g1) |
| *Daphnia pulex* | gnl|ti|884241379 (AZWZ621052.g1) |
