# Supplementary material for: Evolution of a novel subfamily of nuclear receptors with members that each contain two DNA binding domains
Source: BMC Evol Biol. 2007 Feb 23;7:27. doi: 10.1186/1471-2148-7-27 (PMC1810520; doi:10.1186/1471-2148-7-27)
Supplement: Additional File 2 — lists of GenBank accession number of cDNA of human NRs analyzed in this study [file 1471-2148-7-27-S2.doc]

Additional file 2. **GenBank accession number of cDNA of human NRs analyzed in this study**

| Gene name | Accession number |
| --- | --- |
| TRa | X55005 |
| TRb | X04707 |
| RARa | X06614 |
| RARb | X07282 |
| RARg | M24857 |
| PPARa | L07592 |
| PPARb | L02932 |
| PPARg | U79012 |
| Rev-erb-a | M24898 |
| RORa | U04897 |
| RORb | Y08639 |
| RORg | U16997 |
| LXRa | U22662 |
| LXRb | U07132 |
| FXR | U68233 |
| VDR | J03258 |
| PXR | AF061056 |
| CAR | Z30425 |
| HNF4a | X76930 |
| HNF4g | Z49826 |
| RXRa | X52773 |
| RXRb | X63522 |
| RXRg | U38480 |
| TR2 | M29960 |
| TR4 | U10990 |
| TLX | Y13276 |
| PNR | AF121129 |
| COUP-TFI | X16155 |
| COUP-TF-II | M64497 |
| EAR2 | X12794 |
| ERa | X03635 |
| ERb | AB006590 |
| ERRa | X51416 |
| ERRb | AF094517 |
| ERRg | AF094518 |
| GR | X03225 |
| MR | M16801 |
| PR | X51730 |
| AR | M20132 |
| NGFIB | D49728 |
| NURR1 | X75918 |
| NOR1 | D78579 |
| SF1 | U76388 |
| LRH1 | U80251 |
| GCNF | X99975 |
