# Supplementary material for: Evolution of a novel subfamily of nuclear receptors with members that each contain two DNA binding domains
Source: BMC Evol Biol. 2007 Feb 23;7:27. doi: 10.1186/1471-2148-7-27 (PMC1810520; doi:10.1186/1471-2148-7-27)
Supplement: Additional File 3 — lists of GenBank accession number of cDNA sequences of D. melanogaster NRs analyzed in this study [file 1471-2148-7-27-S3.doc]

Additional file 3**: GenBank accession number of cDNA sequences of *D. melanogaster* NRs analyzed in this study**

| Gene name | Accession number |
| --- | --- |
| E75 | X51548 |
| E78 | U01087 |
| DHR3 | M90806 |
| EcR | M74078 |
| DHR96 | U36792 |
| HNF4 | U70874 |
| USP | X53417 |
| DHR78 | U36791 |
| TLL | M34639 |
| PNR | NM_166092 |
| DSF | AF106677 |
| fax-1 | NM_141390 |
| SVP | M28863 |
| ERR | AY051632 |
| DHR38 | X89246 |
| FTZ-F1 | M63711 |
| FTZ-F1b | L06423 |
| GRF | AL035245 |
